# Pancreatic Cancer: Translating Tumor Biology into Actionability

**DOI:** 10.1158/2159-8290.CD-25-2014

**Published:** 2026-07-01

**Authors:** Fiyinfolu O. Balogun, Mara H. Sherman, Wungki Park, Kevin C. Soares, Marsha Reyngold, Joshua D. Schoenfeld, Anupriya Singhal, Christine A. Iacobuzio-Donahue, Eileen M. O’Reilly

**Affiliations:** 1 Department of Medicine, Memorial Sloan Kettering Cancer Center, New York, New York.; 2 David M. Rubenstein Center for Pancreatic Cancer Research, Memorial Sloan Kettering Cancer Center, New York, New York.; 3 Department of Medicine, Weill Cornell Medicine, New York, New York.; 4 Cancer Biology and Genetics Program, Sloan Kettering Institute, Memorial Sloan Kettering Cancer Center, New York, New York.; 5 Department of Surgery, Memorial Sloan Kettering Cancer Center, New York, New York.; 6 Department of Radiation Oncology, Memorial Sloan Kettering Cancer Center, New York, New York.; 7 Department of Pathology and Laboratory Medicine, Memorial Sloan Kettering Cancer Center, New York, New York.

## Abstract

Pancreatic ductal adenocarcinoma (PDAC) accounts for 90% of pancreatic cancers and has a very poor prognosis. Ten to 15% are staged as resectable at diagnosis, and 5% to 15% downstaged with therapy to where surgery is feasible. Chemotherapy is a mainstay for all stages of PDAC. Targeted therapies are available for patients with select but expanding actionable genomic alterations. The tumor microenvironment provides a dense stroma with an immunosuppressive milieu that contributes to inherent treatment resistance of PDAC. Herein, we review current management of PDAC with a focus on emerging treatment paradigms, including targeted and immunomodulatory agents.

**Significance:**

PDAC is a complex disease with unique genomic, immunologic, and clinical features. Recent developments in understanding of the pathobiology of this disease are translating into targeted and immunomodulatory therapies that will alter treatment paradigms and improve outcomes for this recalcitrant malignancy.

## Current Treatment Paradigms

### Early-Stage Disease

Approximately 15% to 20% of PDAC are resectable at diagnosis, with multimodality therapy maximizing the chances of an optimal outcome. Perioperative treatment typically includes 6 months of cytotoxic therapy (Table 1). The CONKO (Charité Onkologie)-001 study identified gemcitabine as a recommended single agent with improvement in median overall survival [mOS; HR, 0.76; 95% confidence interval (CI), 0.61–0.95; *P* = 0.01; ref. 1]. The ESPAC-4 trial established the benefit of multi-agent therapy, with gemcitabine plus capecitabine demonstrating superiority over single-agent gemcitabine (mOS HR, 0.83; 95% CI, 0.71–0.98; *P* = 0.031; ref. 2). The PRODIGE (Partenariat de Recherche en Oncologie Digestive)-24 trial identified FOLFIRINOX [5-fluorouracil (5-FU), oxaliplatin, irinotecan, and leucovorin (LV)] as the standard adjuvant regimen for patients with Eastern Cooperative Oncology Group (ECOG) 0 to 1, demonstrating median disease-free survival (DFS) of 21.6 months compared with 12.8 months in gemcitabine (HR, 0.58; 95% CI, 0.46–0.73; *P* < 0.001; ref. 3). Gemcitabine/nab-paclitaxel was explored as adjuvant therapy in the randomized phase III APACT (Adjuvant Pancreatic Adenocarcinoma Clinical Trial) study, where it was not found to be superior to gemcitabine monotherapy in the primary endpoint of DFS (HR, 0.88; 95% CI, 0.73–1.06; *P* = 0.18; ref. 4). A significant benefit in OS, a secondary endpoint, was noted with 40.5 months for gemcitabine/nab-paclitaxel and 36.2 months for gemcitabine (HR, 0.82; 95% CI, 0.680–0.996; *P* = 0.045). A current standard management strategy for resectable PDAC is initial surgery followed by adjuvant chemotherapy. However, equipoise remains between adjuvant and neoadjuvant therapy in resectable PDAC. PDAC is characterized by early dissemination, even in disease staged as resectable, underpinning a potential benefit for neoadjuvant systemic therapy.

**Table 1.** Current treatment regimens for pancreatic cancer.

Study	Regimen	Control	*N*	Survival outcome
Adjuvant/neoadjuvant				
PRODIGE-24 (3)	mFOLFIRINOX	Gemcitabine	493	3 years DFS: 39.7% vs. 21.4% mOS: 54.4 months vs. 35 months
APACT (4)	Gemcitabine Nab-paclitaxel	Gemcitabine	866	mDFS: 19.4 months vs. 18.8 months; *P* = 0.18 mOS: 41.8 months vs. 37.7 months; *P* = 0.0091
ESPAC-4 (153, 154)	Gemcitabine Capecitabine	Gemcitabine	732	mOS: 30.2 months vs. 27.9 months; *P* = 0.03
NRG/RTOG-0848	Gemcitabine Erlotinib	Gemcitabine	322	mOS: 29.9 months vs. 28.1 months; *P* = 0.062
CONKO-001 (1)	Gemcitabine	Observation	354	mDFS: 13.4 months vs. 6.7 months; *P* < 0.001 mOS: 22.8 months vs. 20.2 months; *P* = 0.01
Metastatic/advanced				
NAPOLI-3 (16)	NALIRIFOX	Gemcitabine Nab-paclitaxel	770	mPFS: 7.4 months vs. 5.6 months; *P* < 0.0001 mOS: 11.1 months vs. 9.2 months; *P* = 0.036
PRODIGE-4 (25)	FOLFIRINOX	Gemcitabine	342	mPFS: 6.4 months vs. 3.3 months; *P* < 0.001 mOS: 11.1 months vs. 6.8 months; *P* < 0.001
MPACT (26)	Gemcitabine Nab-paclitaxel	Gemcitabine	861	mPFS: 5.5 months vs. 3.7 months; *P* < 0.001 mOS: 8.5 months vs. 6.7 months; *P* < 0.001
Maintenance g*BRCA1/2*, *PALB2* (stage III/IV; ref. 28)	Gemcitabine Cisplatin Veliparib	Gemcitabine Cisplatin	50	mPFS: 10.1 months vs. 9.7 months; *P* = 0.73 mOS: 15.5 months vs. 16.4 months; *P* = 0.6
NCIC CTG PA.3 (155)	Gemcitabine Erlotinib	Gemcitabine	569	mPFS: 3.75 months vs. 3.55 months; *P* = 0.004 mOS: 6.24 months vs. 5.91 months; *P* = 0.023
GEM-CAP (156)	Gemcitabine Capecitabine	Gemcitabine	533	mPFS: 5.3 months vs. 3.8 months; *P* = 0.004 mOS: 7.1 months vs. 6.2 months; *P* = 0.08
Gemcitabine vs. fluorouracil (157)	Gemcitabine	Bolus 5-FU	126	mTTP: 9 weeks vs. 4 weeks; *P* = 0.0002 mOS: 5.65 months vs. 4.41 months; *P* = 0.0025
FOLFIRI (158)	FOLFIRI	N/A	63	mTTP: 3 months; 95% CI, 2.1–3.9 mOS: 6.6 months; 95% CI, 5.3–8.1
CapeOx (159)	Capecitabine Oxaliplatin	N/A	41	mOS: 23 weeks; 95% CI, 9.6–14.5
GAP (gemcitabine + nab-paclitaxel + cisplatin; ref. 160)	Gemcitabine Nab-paclitaxel Cisplatin	N/A	25	mPFS: 10.1 months; 95% CI, 6–12.5 mOS: 16.4 months; 95% CI, 10.2–25.3
Gemcitabine Docetaxel Capecitabine (161)	Gemcitabine Docetaxel Capecitabine	N/A	35	*Responders* mPFS: 6.3 months; 95% CI, 4.4–10.4 mOS: 11.2 months; 95% CI, 8.1–15.1

Tumor-agnostic therapies with regulatory approval or guideline endorsement in pancreatic cancer				
Drug	Studies/biomarker	# Tumor types	*N*	Survival outcome
Dabrafenib + Trametinib (30, 162)	*BRAF-V600E*	24	131	ORR: 41%
Entrectinib (31, 163)	*NTRK* fusion+	14	121	ORR: 61.2%
Larotrectinib (164)	*NTRK* fusion+	17	55	
Repotrectinib (165)	*NTRK* fusion+	14	88	ORR, TKI naïve/pretreated: 58/50%
Pembrolizumab (35, 166)	MSI-H/dMMR TMB-H (≥10 Mt/Mb)	27 10	233 790	ORR: 34.3%/mOS: 23.5 months ORR: 30%
Dostarlimab (167)	MSI-H/dMMR	15	327	ORR: 44%
Ipilimumab + Nivolumab (168)	TMB-H (≥10 Mt/Mb)	29	201	ORR, tissue TMB-H: 38.6% ORR, blood TMB-H: 22.5%
Selpercatinib (169)	*RET* fusion	13	41	ORR: 43.9%
Erdafitinib (170)	*FGFR* alterations	31	217	ORR: 30%
Trastuzumab Deruxtecan (34)	*HER2*+	19	267	ORR: 37.1%
Zenocutuzumab (33)	*NRG1* fusion	12	204 (158^a^)	ORR: 30%
Adagrasib (80)	*KRAS-G12C*	9	64 (57^a^)	ORR: 35.1%
Sotorasib (81)^b^	*KRAS-G12C*	1 (Pancreas only)	38	21%

a Efficacy population.

b Not tumor-agnostic.

#### Therapy Sequencing in Early-Stage Disease—Neoadjuvant Versus Adjuvant

Early studies with neoadjuvant chemotherapy indicated a benefit in borderline resectable (BRPC) and locally advanced (LAPC) disease but not resectable disease (5). More recently, PREOPANC compared neoadjuvant gemcitabine with chemoradiotherapy and adjuvant gemcitabine to adjuvant gemcitabine alone in resectable and BRPC disease. This revealed mOS benefit in the neoadjuvant arm (HR, 0.73; 95% CI, 0.56–0.96; *P* = 0.025; ref. 6), evident in both resectable and borderline resectable disease. The neoadjuvant arm also showed improved resection rate (72% vs. 40%; *P* < 0.001), locoregional control, and delay in recurrence. The follow-up PREOPANC-2 trial compared the same neoadjuvant arm with eight cycles of neoadjuvant FOLFIRINOX but showed similar survival (HR, 0.88; 95% CI, 0.69–1.13; *P* = 0.32) and response rate in both arms (7). These, along with other studies, have suggested a benefit of neoadjuvant chemotherapy for resectable disease (8–10), but these trials have mostly used suboptimal comparator chemotherapy arms. The NORPACT-1 trial, a randomized phase II trial, compared neoadjuvant mFOLFIRINOX (four doses neoadjuvant) with adjuvant chemotherapy in resectable PDAC (11). Neoadjuvant mFOLFIRINOX was associated with higher R0 (margin-negative) resection and N0 status at resection; however, the primary endpoint of 18 months OS favored upfront surgery (60% vs. 73%; *P* = 0.032). With compelling arguments both for and against neoadjuvant chemotherapy in resectable disease and no definite evidence of survival benefit, we favor adjuvant therapy for many based on the highest level of evidence but recommend neoadjuvant therapy for patients based on select tumor, biologic, and clinical characteristics. The ongoing PREOPANC-3 (NCT04927780) and ALLIANCE A021806 (NCT04340141) trials will be instrumental in addressing the question of neoadjuvant therapy versus upfront surgery in resectable PDAC as they both utilize current optimal multi-agent chemotherapy (mFOLFIRINOX) in the neoadjuvant and control adjuvant arms.

#### Chemotherapy and Radiotherapy in Borderline/Locally Advanced PDAC

BRPC and LAPC may be downstaged and rendered resectable, improving chances of survival in a subset of patients. Iterative multidisciplinary evaluation should be utilized for this patient subset to adjudicate resectability with treatment over time. The ideal chemotherapy regimen for BRPC/LAPC (Table 1) and whether a role exists for radiotherapy (RT) have not been clearly established. However, a large meta-analysis showed that combination regimens resulted in higher resection rates (5). A large retrospective study that examined FOLFIRINOX as initial treatment for localized PDAC included 958 patients with LAPC and 531 with BRPC (12). The mOS for patients with BRPC was 23.2 months (95% CI, 21–25.7) and 18.7 months for LAPC (95% CI, 17.7–19.9), with resection rates of 53.1% and 17.6%, respectively. PREOPANC showed that perioperative RT concurrent with gemcitabine improved R0 resection rate and OS over adjuvant gemcitabine alone (13). The A021501 phase II randomized trial compared perioperative mFOLFIRINOX with or without RT in BRPC with historic controls (9). The 18-month OS was favorable in the chemotherapy only arm (66.7%, 95% CI, 56.1–79.4) but not in the chemoradiotherapy arm, which closed early (47.3%, 95% CI, 35.8–62.5). ESPAC-5, a small 4-arm randomized phase II trial showed higher 1-year DFS in patients that received neoadjuvant therapy (gemcitabine–capecitabine or FOLFIRINOX or capecitabine-based RT) compared with upfront surgery—59% versus 33%, respectively (HR, 0.53; 95% CI, 0.28–0.98; *P* = 0.016). One year OS in the neoadjuvant arm was 76%, versus 39% in the upfront surgery arm (HR, 0.29; 95% CI, 0.14–0.60; *P* = 0.0052; ref. 8). The Japan Clinical Oncology Group 1407 study showed that both neoadjuvant FOLFIRINOX and gemcitabine/nab-paclitaxel arms in patients with LAPC performed better than historic controls but were not designed to be compared directly (14). A retrospective study by the Trans-Atlantic Pancreatic Surgery consortium investigated the benefit of adding RT to neoadjuvant chemotherapy in the treatment of patients with BRPC (15). The mOS was 26.2 months (95% CI, 24–34.8) in the RT arm and 32.8 months (95% CI, 25.3–42) in the non-RT arm; no significant difference was seen (*P* = 0.71). Although the RT arm had a lower resection rate, it was associated with more node-negative disease (57.3% vs. 37.6%; *P* = 0.002) and higher pathologic response (24.7% vs. 8.3%; *P* = 0.006) at resection (15). As NALIRIFOX (liposomal irinotecan, 5-FU, LV, and oxaliplatin) has shown superiority over gemcitabine/nab-paclitaxel in the metastatic setting (NAPOLI-3; ref. 16), a single-institution study is investigating NALIRIFOX sequenced with ablative dose RT (NCT05851924) in BRPC/LAPC as part of a total neoadjuvant therapy paradigm.

The role of RT in PDAC is evolving. Use of conventional radiation dosing paradigms has not shown definitive OS benefit in any disease setting of PDAC (17). Early studies showed a trend toward improved OS with adjuvant chemoradiotherapy over surgery alone; however, these trials were notable for small sample size, study design, quality control, and compliance limitations (Gastrointestinal Tumor Study Group and European Organization for Research and Treatment of Cancer; ref. 18). The NRG (National Radiation Group)/RTOG (Radiation Therapy Oncology Group)-0848 phase III trial in resected pancreas head cancer presented at the 2024 American Society of Clinical Oncology (ASCO) conference showed improved DFS (HR, 0.82; 95% CI, 0.68–0.99; *P* = 0.045) but no OS benefit with the addition of RT to a primarily gemcitabine-based adjuvant chemotherapy regimen, final manuscript in review (19). In patients with locally advanced PDAC whose disease had not progressed after 4 months of gemcitabine (±erlotinib), chemoradiotherapy decreased rates of local recurrence (32% vs. 46%, *P* = 0.03) and improved progression-free survival (PFS; 6.1 vs. 3.7 months, *P* = 0.02) versus continued chemotherapy. Although there was no OS benefit observed, RT allowed for a longer duration without treatment, potentially improving quality of life (20). For decades, multiple studies have failed to demonstrate significant benefit from this modality in PDAC; however, newer approaches may change this understanding. Emerging techniques such as “ablative” radiation approaches provide up to twofold higher biologically equivalent dosing relative to conventional dosing and have shown potential for improved localized control as identified in select institutional experiences, including as a definitive treatment option in medically unfit patients with resectable disease (21–23). Ablative dose RT has increasingly been used in the BRPC/LAPC setting and is reported in single-institution studies to be more beneficial than standard dose RT but requires evaluation in larger randomized studies (22). Further clarification is expected from the ongoing randomized phase III trial comparing ablative dose RT with standard of care in LAPC (NCT06958328). The use of electric fields (tumor treating fields, TTF) combined with gemcitabine/nab-paclitaxel was studied in the randomized phase III PANOVA-3 study that showed a modest improvement in OS (HR, 0.82; 95% CI, 0.68–0.99; *P* = 0.039) with no increase in significant toxicity, spurring the potential of using TTF in multimodality therapeutic regimens including RT (24).

### Metastatic Disease

Chemotherapy is the staple for advanced PDAC (Table 1). For patients with an ECOG performance status of 0 to 1, multi-agent chemotherapy regimens such as (modified; dose-adjusted) mFOLFIRINOX (PRODIGE-4 Trial—HR, 0.57; 95% CI, 0.45–0.73; *P* < 0.001) and gemcitabine/albumin-bound (nab) paclitaxel (MPACT, Metastatic Pancreatic Adenocarcinoma Clinical Trial—HR, 0.72; 95% CI, 0.62–0.83; *P* < 0.001) improve survival over single-agent gemcitabine (25, 26). In the NAPOLI-3 trial, NALIRIFOX improved OS over gemcitabine/nab-paclitaxel (HR, 0.84; 95% CI, 0.71–0.99; *P* = 0.04; ref. 27). Homologous recombination–deficient (HRD) tumors with mutations in *BRCA1/2* and *PALB2* are particularly sensitive to platinum-based chemotherapy (28), and thus first-line regimens in this setting should include a platinum agent. In the Pancreatic Adenocarcinoma Signature Stratification for Treatment (PASS-01) trial, noting the exclusion of patients with *BRCA/PALB2* mutant tumors and discussed in detail below, FOLFIRINOX had worse OS compared with gemcitabine/nab-paclitaxel in first line for metastatic PDAC (HR, 1.57; 95% CI, 1.08–2.28; *P* = 0.017; ref. 29). This raises a question of subtype susceptibility to different chemotherapy regimens and highlights the need for biomarker identification. Targeted agents approved for use in PDAC include dabrafenib and trametinib combination in *BRAF-V600E* tumors (30), larotrectinib and entrectinib for tumors with *NTRK* gene fusions (31, 32), and zenocutuzumab for *NRG1* fusion tumors (33). Trastuzumab deruxtecan was approved for tumor-agnostic HER2-expressing tumors (34). Immune checkpoint blockade (ICB) with tumor-agnostic approval is relevant to the 1% of PDAC that are mismatch repair–deficient (dMMR)/microsatellite instable (MSI-H) or have tumor mutational burden (TMB) >10 mutations/megabase (35). Second-line treatments include fluoropyrimidine-based therapy for disease that progressed on gemcitabine-based therapy and *vice versa*. In NAPOLI-1, liposomal-irinotecan combined with 5-FU/LV improved mOS to 6.2 months compared with 4.2 months in 5-FU/LV for patients whose disease progressed on prior gemcitabine-based therapy (HR, 0.75; 95% CI, 0.57–0.99; *P* = 0.037; Table 1; ref. 36).

## Advancement of Current Regimens

### Therapy Sequencing in Metastatic PDAC

Recent studies on current chemotherapy regimens are exploring the most effective treatment sequence. The SEQUENCE trial reported that gemcitabine/nab-paclitaxel alternated with FOLFOX (5-FU, oxaliplatin, and LV) improved 12 month OS (55.3% vs. 35.4%, *P* = 0.02) and mOS (13.2 months vs. 9.7 months; HR, 0.068; 95% CI, 0.48–0.95) compared with gemcitabine/nab-paclitaxel alone, with higher toxicity (37). The GABRINOX phase I/II trial showed that alternating gemcitabine/nab*-*paclitaxel with FOLFIRINOX is promising, with overall response rate (ORR) 64.9%, PFS 10.5 months, and OS 15.1 months (38). The follow-up GABRINOX-2 is an ongoing randomized phase II trial (NCT05065801) to compare with a FOLFIRINOX arm (39). The FOOTPATH study evaluated alternating NAPOLI (liposomal irinotecan, 5-FU, and LV) with FOLFOX versus NAPOLI versus gemcitabine/nab-paclitaxel. Neither NAPOLI/FOLFOX nor NAPOLI improved PFS or OS over standard-of-care gemcitabine/nab-paclitaxel (40).

### Localized Therapy in Metastatic PDAC

The role of local therapy in metastatic disease is evolving and debated. Oligometastatic disease with potential for localized intervention to metastatic sites is treated primarily with systemic therapy, reserving localized interventions such as RT for palliative management of symptoms. Retrospective studies that mostly defined oligometastatic disease as three or less metastatic sites suggest a benefit of localized metastatic therapy, with improvement in survival (41). However, these studies are unable to determine whether the positive findings are a benefit of localized interventions or a reflection of patient selection and disease biology (42). The results from the phase II EXTEND (external beam radiation to eliminate nominal metastatic disease) showed benefit of metastases-directed RT by delaying disease progression and extending PFS (43). Patients with less than six metastatic sites were randomized to systemic therapy ± metastases-directed therapy (MDT). PFS was higher in the MDT + systemic therapy arm at 10.3 months versus 2.5 months in the systemic only arm (HR, 0.43; 95% CI, 0.20–0.94; *P* = 0.03). A follow-up phase III trial (EXPAND) is underway to fully understand the role of local therapy integrated with systemic therapy in metastatic disease (NCT06593431). Ongoing trials in liver-limited oligometastatic PDAC include HOLIPANC (hepatic oligometastatic adenocarcinoma of the pancreas) that combines chemotherapy with hepatectomy (NCT04617457); CSPAC (Chinese Study Group for Pancreatic Cancer)-1, a randomized trial to study the survival benefit of hepatectomy (NCT03398291); and SCANPAN (Scandinavian Pancreas Group)-1, a prospective cohort study to evaluate hepatectomy (NCT05271110). Another option for therapy that is fast gaining traction is high-intensity focused ultrasound (HIFU), thus far largely limited to liver metastatic disease but with ongoing exploration of its role in primary disease (44). HIFU is a noninvasive thermal ablation technique that causes coagulative necrosis in the treatment of tumors.

### Maintenance Therapy

Maintenance therapy intent is to decrease toxicity without compromising survival by deintensification of treatment in patients with advanced disease. Several studies have suggested that deintensification of chemotherapy in PDAC reduces toxicity without affecting survival (45). The randomized phase II ALPACA study compared a treatment arm alternating gemcitabine/nab-paclitaxel and gemcitabine monotherapy to a continuous gemcitabine/nab-paclitaxel arm in patients with metastatic PDAC. The results noted similar OS (10.5 vs. 10.4 months; HR, 0.90; 80% CI, 0.72–1.13; *P* = 0.56) and PFS (5.5 vs. 5.4 months; HR, 0.80; 95% CI, 0.58–1.11; *P* = 0.18). Notably decreased peripheral neuropathy in the alternating arm of 62%, compared with 74% in the continuous arm, indicated potential benefit of increased quality of life (46). Targeted agents and immunotherapy also play a role in maintenance therapy, with poly (ADP-ribose) polymerase inhibitors (PARPi) representing a chemotherapy-sparing option for patients with germline *BRCA/PALB2*-mutated PDAC that respond to platinum agents (47, 48). The phase III Pancreas OLaparib Ongoing (POLO) trial demonstrated that olaparib administered for platinum-sensitive, germline *BRCA1/2*-mutant tumors improved PFS to 7.4 months in the olaparib arm versus 3.8 months in the placebo arm (HR, 0.53; 95% CI, 0.35–0.82; *P* = 0.004; ref. 48). A single-arm phase II study of rucaparib maintenance in patients with platinum-sensitive germline/somatic *BRCA1*/2 or *PALB* tumors yielded a PFS of 13.1 months (95% CI, 4.4–21.8) and OS of 23.5 months (95% CI, 20–27; ref. 47). Initial results from the PARPVAX trial (NCT03404960) showed that combining a PARPi (niraparib) with ICB (nivolumab or ipilimumab) in platinum-sensitive PDAC is safe and active (49). The recently completed Pembrolizumab and OLapARib (POLAR) trial combined ICB (pembrolizumab) with PARPi (olaparib) in patients with PDAC tumors according to distinct groups: HRD tumors (cohort A), non-core HRD tumors (cohort B), and non-HRD tumors with exceptional platinum response (cohort C; ref. 50). Interim results presented at the 2024 ESMO Congress showed median PFS (mPFS) in cohort A of 8.2 months, cohort B 4 months, and cohort C 3.3 months. OS also favored cohort A (A – NR; B – 18 months; C – 10 months), with final clinical and molecular analysis pending in the upcoming manuscript. The TEDOPam study (NCT03806309) is a randomized phase II trial evaluating FOLFIRI ± OSE2101 (a vaccine targeting five tumor antigens frequently expressed in PDAC) in the maintenance setting after FOLFIRINOX. Interim results presented at the 2025 ASCO conference showed favorable OS in both arms with no significant difference in the primary end point of 12 months OS (FOLFIRI/OSE2101 – 65%; FOLFIRI – 61%; ref. 51). An ongoing immunotherapy-based maintenance trial randomizes patients to domvanalimab (anti-TIGIT)/zimberelimab (anti–PD-1)/APX005M (CD40 agonist) versus FOLFIRI as maintenance after disease control with 4 to 6 months FOLFIRINOX (NCT05419479). This study has closed enrollment with the results pending.

## Emerging Targeted Therapies

### Evaluating Genomic and Transcriptomic Descriptors in PDAC

Growing understanding of the genomic and molecular basis of PDAC has led to an increase in novel targeted therapies that improve efficacy and decrease toxicity. Mutational signatures have been identified to correlate with different etiologies of PDAC. Established driver oncogenes in PDAC include *KRAS*, *CDKN2A*, *TP53*, and *SMAD4*. About 3.8% to 9.7% of patients with PDAC have a pathogenic germline mutation, most commonly in *BRCA2*, *BRCA1*, and *ATM*, with *BRCA1/2* and *PALB2* representing “core” genes involved in homologous repair. Other less common genes contributing to germline predisposition include *TP53*, *STK11*, *APC*, *PRSS1*, *CDKN2A*, and mismatch repair genes (*MLH1*, *MSH2*, *MSH6*, *PMS2*, and *EPCAM*; refs. 52–54). Whole-genome and transcriptomic analyses of PDAC have led to classification of the disease into molecular subtypes including “classic” and “basal”, with therapeutic implications (55).

The increasing breadth of knowledge and role of genomics in therapeutic decision making in PDAC adds significant complexity to clinical management and trial design. To facilitate this, molecular-based disease management group conferences (molecular tumor boards, MTB) are being piloted as a part of clinical trials such as the PASS-01 trial (NCT04469556). PASS-01 is a randomized phase II trial between FOLFIRINOX and gemcitabine/nab-paclitaxel with extensive molecular tissue and blood testing to identify biomarkers that can guide treatment options for patients with metastatic PDAC. In addition, patient-derived organoids (PDO) are obtained and treated with multiple drugs to assess for drug sensitivity. Regular MTBs are held to analyze data for each patient that includes clinical, genomic, PDO screen, and literature review to arrive at a treatment recommendation for the patient’s provider. The use of sequencing and PDO data to guide second-line therapy had a survival of 5.4 months versus 4.4 months when using standard chemotherapy (*P* = 0.45; ref. 29). Most importantly, it demonstrated the feasibility of incorporating real-time multiomic profiling, including PDO drug screening into clinical care guided by MTBs. In a real-world study to illustrate the benefit of these conferences, an MTB reviewed next-generation sequencing, RNA expression, and immunohistochemistry data in patients with various malignancies (pancreatic being the fifth most common) and then provided matched therapy recommendations to their primary physicians (56). Of the 429 evaluable patients, 62% were matched to at least one MTB recommended drug (20% matched to all MTB recommended drugs), whereas 38% received physician choice regimen. Survival was improved in patients matched to all MTB recommended drugs compared with physician choice regimen in both PFS (HR, 0.68; 95% CI, 0.51–0.90; *P* = 0.008) and OS (HR, 0.69; 95% CI, 0.49–0.98; *P* = 0.036). Furthermore, patients that received high (≥50% match of alterations) versus low (<50% match of alterations) matching score therapy had improved PFS (HR, 0.63; 95% CI, 0.50–0.80; *P* < 0.001) and OS (HR, 0.67; 95% CI, 0.50–0.90; *P* = 0.007).

### Biomarker-Driven Treatment

Personalized precision medicine driven by biomarker-guided therapies is widely established as a therapeutic paradigm in cancer management, including in select patients with PDAC. A retrospective study in PDAC evaluating biomarker-matched therapy demonstrated improved OS in patients with actionable variants compared with those without actionable variants or receipt of unmatched therapy (57). Approved genetic biomarkers include HRD determinants (*BRCA/PALB2*) as pancreas-specific indication, whereas dMMR/MSI-H, TMB, *NTRK*, *BRAF-V600E*, *RET*, and *NRG1* represent targets with tumor-agnostic regulatory approval. Another potential biomarker is tumor phenotype, previously introduced, for which classifications such as basal and classic subtypes may predict therapy response (55, 58). These subtypes partially align with specific pathways and have the potential to serve as biomarkers for treatment selection. Findings from the PASS-01 trial, discussed above, suggested survival benefit with biomarker-driven selection of chemotherapy, including guided chemotherapy choice based on “classic” and “basal” subtypes (29). The phase II RAGNAR study is a basket trial that treated patients with *FGFR*-altered tumors with erdafitinib, a pan-FGFR tyrosine kinase inhibitor. In the subset of patients (*N* = 18) with previously treated metastatic PDAC, clinical activity was observed with ORR 55.6% (95% CI, 30.8–78.5) and mOS 19.7 months (59).

### Novel Biomarkers and Testing

Blood-based assays that assess DNA have demonstrated prognostic ability across several gastrointestinal cancer types (60). In PDAC, a chemosensitivity assay based on gene expression profiles was shown to be predictive of response to first-line chemotherapeutic regimens (61). Patients predicted to be sensitive had clinically significant increase in PFS (HR, 0.35; *P* = 0.002) and OS (HR, 0.40; *P* = 0.005) over those predicted to be resistant. Novel biomarker approaches being studied to guide therapy selection include immune-based selection tools such as neutrophil-to-lymphocyte ratio, which seems predictive for treatment with gemcitabine and CD-40 agonist (62); tissue factor testing (NCT04843709); and CLDN18.2 to predict response to claudin 18.2 antibody (NCT03816163). Claudin 18.2 expression seems to be associated with better prognosis; however, some studies indicate it is associated with poorer prognosis or independent of prognosis (63). Claudin 18.2 is not detected in normal pancreas tissue but found expressed in pancreatic cancer, making it a good target for diagnosis and treatment of PDAC. Zolbetuximab targets CLDN18.2 and has shown survival benefit when combined with chemotherapy in the first-line treatment of gastric/gastroesophageal cancer, another malignancy associated with increased CLDN18.2 expression (64, 65). Final data analysis from a randomized study of zolbetuximab combined with gemcitabine/nab-paclitaxel in patients with advanced PDAC (NCT03816163) was noted in an October 2025 press release to have not met its primary OS endpoint, final publication pending. One epigenetic pathway of interest involves the interaction between methylthioadenosine phosphorylase (MTAP) and protein arginine methyltransferase 5 (PRMT5). Methylthioadenosine (MTA), the substrate for MTAP, accumulates in *MTAP*-deleted tumors, leading to inhibition of PRMT5, which plays an important role is several cellular processes that promote tumor survival. Homozygous deletion of *MTAP* in pancreas cancer cells is associated with a poor prognosis and leads to tumor cell dependency on PRMT5, thereby presenting a potential target for synthetic lethality (66). AMG-193, a PRMT5 inhibitor, has shown in early studies to be effective in the treatment of pancreatic cancer (67), with ongoing studies to combine it with chemotherapy in the treatment of *MTAP*-deleted tumors (NCT06360354). MRTX1719 selectively inhibits PRMT5 bound to MTA and has demonstrated antitumor activity in ongoing early-phase studies (NCT05245500; ref. 68). Both AMG-193 and MRTX1719 are second-generation inhibitors that are MTA-cooperative, making them selective for *MTAP*-deleted PDAC tumors. Other MTA-cooperative inhibitors in early/mid-phase studies include TNG462 (vopimetostat; NCT05732831), with a planned phase III in second-line PDAC, TNG456 (NCT06810544), BMS-986504 (NCT05245500), and AZD3470 (NCT06130553).

### Antibody-Based Biologics

Antibody–drug conjugates (ADC) contain a tumor-specific/preferred antibody and cytotoxic payload, designed to target deposition of drugs to tumors while minimizing systemic toxic effects. The DESTINY-PanTumor02 phase II trial demonstrated efficacy of trastuzumab deruxtecan in multiple HER2-expressing tumor types, including PDAC, resulting in a tumor-agnostic approval (34). Targeting HER2 is a rationale strategy, as increased phosphorylation observed upon inhibition of the MAPK pathway suggests a tumor-favored alternate pathway that can be effectively targeted (69). Preclinical studies showed benefit when ADC constructs combine KRAS inhibition with HER targeting (69). Several other targets of interests include CLAUDIN 18.2, TROP-2, CA19-9, and tissue factor (70). Recently presented updates for a basket trial of the anti–tissue factor ADC, MRG004A, demonstrated safety and antitumor activity in several malignancies, including pancreatic cancer (NCT03941574; ref. 71).

Bispecific antibodies target two independent antigens simultaneously, and they function through several mechanisms. Zenocutuzumab, a HER2–HER3 bispecific antibody that blocks HER2–HER3 dimerization and the interaction between HER3 and NRG1/NRG1 fusion protein, was recently approved in *NRG1* fusion–positive PDAC (69). Another bispecific design replaces the cytotoxic payload of ADCs with an antibody directed to cytotoxic immune cells. Here, the mechanism of antitumor action is the proximity of these cytotoxic immune cells to the tumor cells, resulting in immune-mediated cell death. ASP2138 is a bispecific antibody to CLDN18.2 and CD3 that showed cytotoxic activity against gastric and pancreatic cancer cells (72). There is an ongoing phase I clinical trial of ASP2138 in patients with pancreatic or gastric/gastroesophageal junction cancer (NCT05365581). Anti-EGFR and anti-CD3 bispecific antibody armed T cells (BAT) were shown to be safe and induced antitumor immune response (73). Twenty patients across phases I and II received BATs for locally advanced and metastatic pancreatic cancer. The mOS for all patients was 14.5 months (95% CI, 7.5–45.2 months) and 31 months for 17 evaluable patients (received at least 75% of infusion in phase I or 100% of infusion in phase II; ref. 74).

### Radioligand Therapies

A growing area of investigation involves the field of radioligand therapy, which combines radioactive molecules with targeting ligands in the treatment of cancer. Novel radiotracers being explored to predict response include the use of a specialized tracer, 18F-FAC, to assess uptake of gemcitabine by the tumor, thereby monitoring the efficacy of chemotherapy access (NCT05141643). Theranostic agents that can both identify and treat malignancies have been established in the management of several diseases, including neuroendocrine and prostate cancers. A key aspect to theranostics is identifying appropriate targets for the radioligand. Cancer-associated fibroblasts (CAF) are increased in the tumor microenvironment and play an important role in promoting tumor progression, making them a promising therapeutic target. CAFs express a peptidase, fibroblast activation protein (FAP), that is associated with poorer prognosis and recently became targetable with radiotracers such as gallium-68 (75, 76). FRONTIER is a multicenter trial that aims to predict response of FAP-overexpressed tumors to [Lu-177]-PN6555 therapy (NCT05432193). Another phase I trial targeting FAP in PDAC pairs gallium Ga 68-DOTA-5G and lutetium Lu 177-DOTA-ABM-5G as a novel theranostic approach (NCT04665947). Several other ongoing trials utilize FAP-targeted radiotracers to identify poor prognosis PDAC and subsequently treat in a highly targeted manner (NCT05262855; NCT04939610).

### *KRAS*-Mutant PDAC

**Figure 1.**
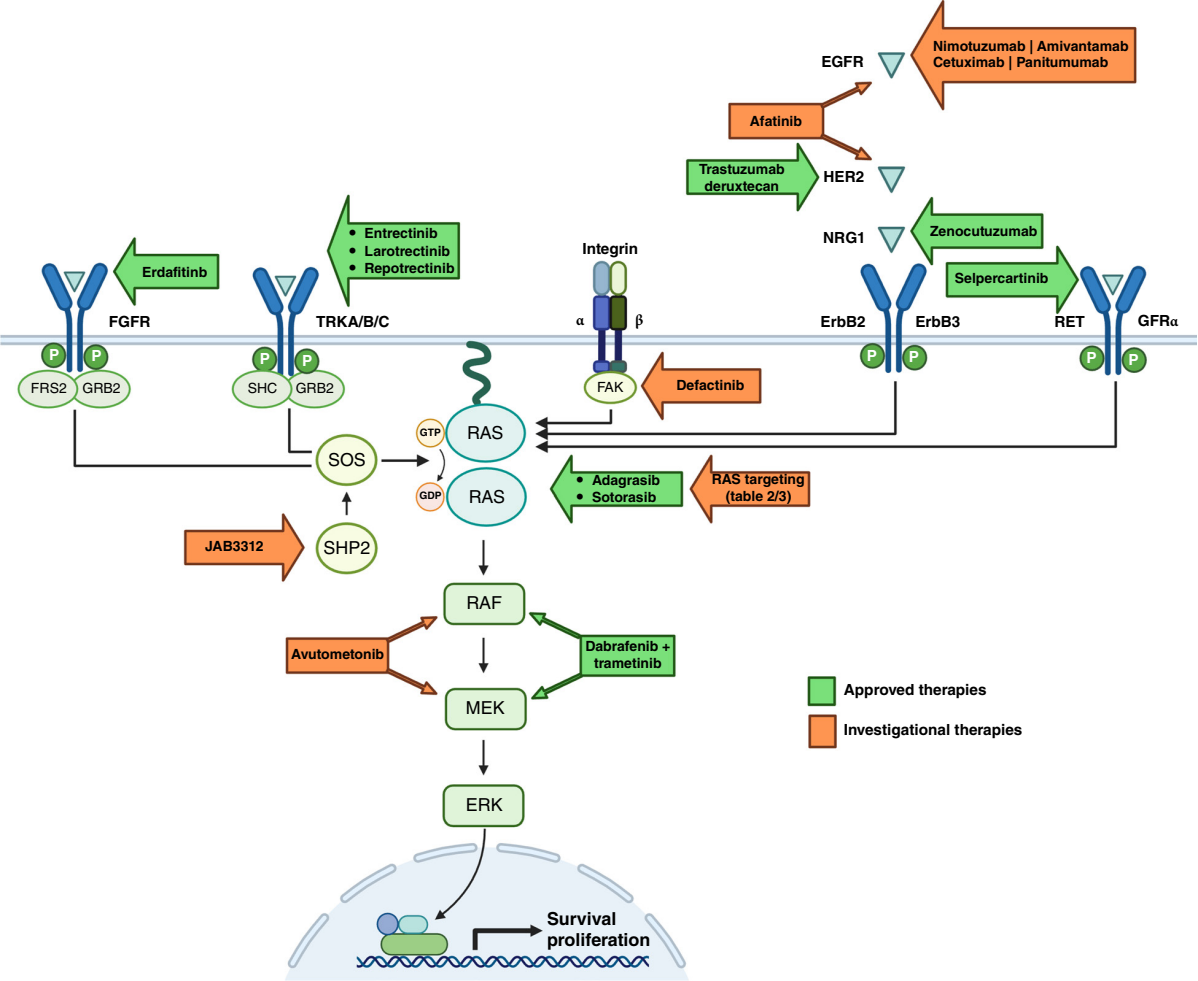
MAPK pathway targeting for pancreatic cancer. [Created in BioRender. Rubenstein, D. (2025) https://BioRender.com/27s6pcf.]

*KRAS* is the most altered oncogenic protein in human cancers, leading to cell proliferation and other tumor-promoting activities (77). Nearly 95% of PDAC harbor a driver mutation in *KRAS*, making PDAC the most RAS-dependent malignancy and representing an attractive therapeutic target (Fig. 1; ref. 78). Copy-number variations of *KRAS*-mutant alleles have the potential to be prognostic, with gain associated with worse outcome (79). KRAS is active when bound to guanosine triphosphate (GTP) and inactive when bound to guanosine diphosphate (GDP), with cycling between these two states regulated by guanine nucleotide exchange factors and GTPase-activating proteins. *KRAS* driver mutations in PDAC result in a constitutively active protein. KRAS was heretofore deemed undruggable; however, the successful targeting of *KRAS-*G12C led to regulatory approval in non–small cell lung cancer and to National Comprehensive Cancer Network guideline inclusion of KRAS-G12C inhibitors (adagrasib and sotorasib) for PDAC, thereby changing this now dated understanding. In the phase II KRYSTAL-1 study, adagrasib resulted in PFS of 5.4 months and ORR of 33.3% (95% CI, 14.6–57) in patients with PDAC whose disease had progressed on an average of 2.5 lines of prior therapy (80). Sotorasib showed PFS of 4 months (95% CI, 2.8–5.6) and ORR of 21% (95% CI, 10–37) in the CodeBreaK phase I and II trials (81). Subsequent generations of drugs and use in earlier lines seem to improve efficacy. The results from two phase I/II trials of glecirasib, a second-generation KRAS-G12C inhibitor, showed higher response when given earlier, with ORR 46.9% (95% CI, 29.1–65.3), mPFS 5.5 months, and mOS 10.8 months in patients with PDAC that received a median of one prior line of therapy (82). Notwithstanding, *KRAS*-G12C accounts for 1% to 2% of PDAC; with G12D, G12V, and G12R being the most common *KRAS*-mutant allele variants observed. *KRAS*-mutant tumors exhibit clinical differences that have been correlated with unique biochemical characteristics identified in the various mutant alleles (83). KRAS-G12C inhibitors such as adagrasib and sotorasib function by forming covalent bonds dependent on a specific cysteine that are not present in other *KRAS*-mutant alleles (77). Consequently, other strategies are underway to target non–*KRAS*-G12C alleles in PDAC (84). Mutant allele-specific KRAS inhibitors increase target specificity; however, this level of specificity also renders the inhibitors potentially more susceptible to resistance.

Sotorasib and adagrasib function by trapping KRAS in the “off” GDP-bound state. Drug designs that target KRAS in the “on” state typically function to inhibit signaling from the activated protein. An example of an “on” state design are the tricomplex inhibitors, which function like a “molecular glue” that binds RAS to a ubiquitous intracellular membrane protein, cyclophilin A, thereby inhibiting guanine exchange and subsequent downstream signaling. The tricomplex’s mechanism of steric hindrance to obstruct signaling allows it to potentially overcome genetic mechanisms of acquired resistance to other allele-specific inhibitors (84, 85). There are many KRAS-targeted drugs at various levels of development. Examples of drug mechanisms include mutant-specific inhibitors (RMC-9805, G12D inhibitor; Table 2), degraders (ASP3082), peptide vaccines (ELI002-2P/7P, V941), T-cell therapies, siRNA, and exosomal approaches under development. Daraxonrasib (RMC-6236; Table 3) uses a unique tricomplex strategy of targeting active KRAS that was shown in phase I/II studies to be well tolerated with promising antitumor activity (86). A subsequent phase III trial, RASolute 302 (NCT06625320), has completed accrual comparing daraxonrasib with investigator’s choice chemotherapy in a second-line RAS mutant and wild-type (WT) PDAC. A press release on 04/13/2026 indicated that all primary and secondary endpoints were met at the time of first and final analysis and detailed data are anticipated at the 2026 ASCO conference (https://ir.revmed.com/news-releases/news-release-details/daraxonrasib-demonstrates-unprecedented-overall-survival-benefit). Specifically daraxonrasib demonstrated a median OS of 13.2 months versus 6.7 months for chemotherapy, HR 0.4, *P* < 0.0001. A pan-KRAS strategy utilized in BI-2865 binds to the inactive form of WT and mutant KRAS, blocking nucleotide exchange and thereby inhibiting downstream signaling (87). A unique approach is the siG12D-LODER, a biodegradable polymeric matrix with siRNA targeting KRAS-G12D/V that is directly inserted into the pancreas via endoscopy (88). Safety of siG12D-LODER was established when combined with chemotherapy in localized disease LAPC in a two-cohort phase II trial with 59 patients. Combination of siG12D-LODER with chemotherapy was well tolerated and generally had toxicities as would be expected with chemotherapy only, the exception being more sepsis in the combination groups, suspected related to the endoscopy placement procedure (88). Survival and response were not significantly different; however, a trend toward improvement in the combination arms suggests further exploration is required.

**Table 2.** *RAS* allele-specific targeted trials in pancreatic cancer—select completed and ongoing.

Study/sponsor	NCT number	Phase	KRAS target	Drug	Mechanism	Status
KontRASt-01 Novartis	NCT04699188	1b/2	G12C	JDQ443 (opnurasib)	Off state inhibitor	Ongoing
Jacobio Pharmaceuticals	NCT05288205	1/2a	G12C	JAB-21822 (glecirasib)	Off state inhibitor	Ongoing
Eli Lilly	NCT04956640		G12C	LY3537982 (olomorasib)	On state inhibitor	Ongoing
PROSPER Frontier Medicines	NCT06244771	1/2	G12C	FMC-376	On/off state inhibitor	Ongoing
D3 Bio	NCT05410145	1	G12C	D3S-001	Off state inhibitor–rapid target engagement	Ongoing
Genentech	NCT04449874	1	G12C	GDC-6036 (divarasib)	Off state inhibitor	Ongoing
KANDLELIT-001 Merck	NCT05067283	1	G12C	MK-1084	Off state inhibitor	Ongoing
InventisBio Co	NCT04585035	1/2	G12C	D-1553 (garsorasib)	Off state inhibitor	Enrollment complete
Revolution Medicines	NCT06040541	1	G12D	RMC-9805	Tricomplex RAS (ON) inhibitor	Ongoing
	NCT06128551	1b	G12C	RMC-6291	Tricomplex RAS (ON) inhibitor	Ongoing
Astellas Pharma	NCT05382559	1	G12D	ASP3082	Targeted protein degrader	Ongoing
GenFleet Therapeutics	NCT07026916	2	G12D	GFH-375	On–off dual state inhibitor	New
Incyte	NCT06179160	1	G12D	INCB161734	Off state inhibitor	Ongoing
MOONRAY-01 Eli Lily	NCT06586515	1	G12D	LY3962673	Off state inhibitor	Ongoing
Quanta Therapeutics	NCT06428500	1	G12D	QTX-3046	Off state inhibitor	Ongoing
Mirati Therapeutics	NCT05737706	1/2	G12D	MRTX1133	Off state inhibitor	Terminated
Jiangsu HengRui	NCT05533463	1	G12D	HRS-4642	Off state inhibitor	Enrollment complete
PROTACT Silenseed	NCT01676259	2	G12D	siG12D-LODER	siRNA	Enrollment complete
Tyligand Pharmaceuticals	NCT06385925	1/2	G12D	TSN-1611	Off state inhibitor	Ongoing
Qilu Pharmaceutical	NCT06403735	1	G12D	QLC-1101	Reversible inhibitor	Ongoing

**Table 3.** *RAS* multi-allele and immune-targeted trials in pancreatic cancer—select completed and ongoing.

Study/sponsor	NCT number	Phase	KRAS target	Drug	Mechanism	Status
Revolution Medicines	NCT05379985	1	Pan-RAS	RMC-6236	—	Ongoing
	NCT06445062	1b/2	Pan-RAS	RMC-6236 + chemotherapy	—	
	NCT06625320	3	Pan-RAS	RMC-6236 (vs. chemotherapy)	—	
AMPLIFY-201 Elicio Therapeutics	NCT04853017	1	G12D, G12R	ELI-002	KRAS peptide vaccine	Ongoing
AMPLIFY-7P Elicio Therapeutics	NCT05726864	1/2	G12D/R/V/A/C/S; G13D	ELI-002 7P	KRAS peptide vaccine	Ongoing
Astellas Pharma	NCT07094204	1	Pan-RAS	ASP5834	Targeted protein degrader	Ongoing
Boehringer Ingelheim	NCT06056024		G12V WT amplification	BI-3706674	Off state, Pan-KRAS	Enrollment complete
Moderna/Merck	NCT05013216	1	G12D/R/V/A/C/D	Pooled KRAS peptide vaccine + ipilimumab/nivolumab	KRAS peptide vaccine + Poly ICLC adjuvant	Enrollment complete
Boehringer Ingelheim	NCT03745326	1	KRAS/HRAS/NRAS G12D TCR HLA-A*11:01 restricted	Anti-KRAS G12D mTCR	CAR T-cell therapy	Ongoing
IMMUNEERING Immuneering Corporation	NCT05585320	1/2a	Pan-RAS (MEK/ERK)	IMM-1-104	MEK/ERK inhibitor	Enrollment complete
GenFleet Therapeutics	Preclinical		Pan-RAS	GFH547	On–off dual state inhibitor	Ongoing
Revolution Medicines	Preclinical		Pan-RAS	RMC-7977	Tricomplex RAS (ON) inhibitor	Ongoing

**Figure 2.**
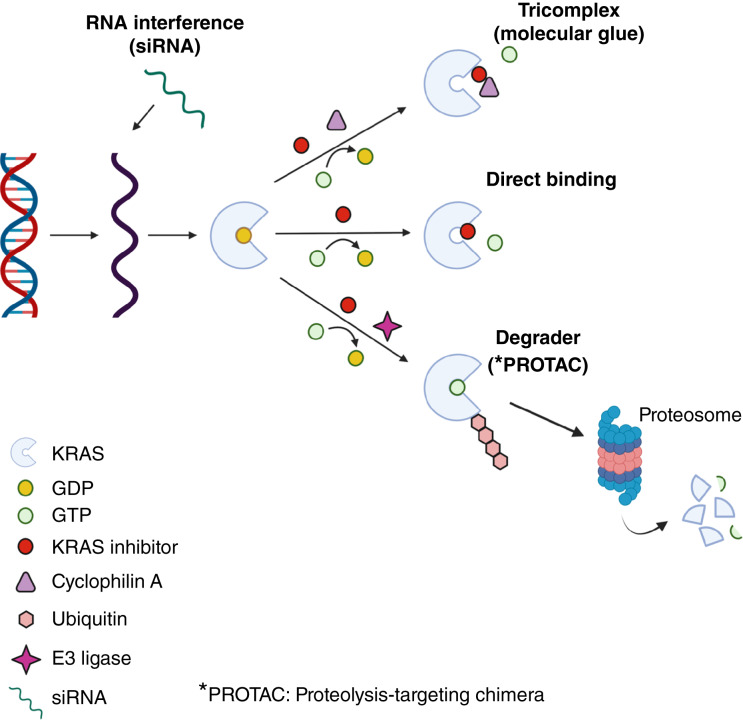
Mechanisms of KRAS inhibitor/degrader.

Resistance to RAS therapeutics is driven by several mechanisms that frequently lead to reestablishment and enhanced activation of the RAS−MAPK signaling pathway (84). These mechanisms include intratumoral heterogeneity resulting in generation of new *RAS* mutations, increased activation of KRAS-WT by upstream kinases, epithelial-to-mesenchymal transition (EMT), bypass genetic mutations, and KRAS-independent activation of the PI3K−AKT pathway (Fig. 2; ref. 89). For example, in *KRAS*-G12C tumors, some of the already identified mechanisms include overriding alterations in *KRAS* or downstream genes, amplification of *KRAS*-G12C or *MET*, and loss-of-function mutations in tumor-suppressor genes (90). Pharmacologic alteration and increased drug efflux are other mechanisms that are applicable to most targeted drugs and may also contribute to RAS inhibitor resistance. *KRAS*-altered tumors typically use multiple resistance mechanisms; therefore, inhibitors designed to overcome resistance will need to target multiple mechanisms. Intelligent use of combinatorial and sequential regimens that include KRAS inhibition will be a key tool in the quest to overcome resistance. This field of KRAS inhibitor resistance has rapidly emerged as a critical area of focus.

Combination regimens that include KRAS targeting is an area of active development to improve treatment response and overcome resistance. To address feedback reactivation as a resistant mechanism to KRAS inhibition, multiple levels in the RAS pathway are being cotargeted in ongoing studies (Fig. 1). One example is EGFR, an effective target in KRAS-WT PDAC (91) that is currently being combined with KRAS inhibition in other malignancies (KRYSTAL-10 NCT04793958 – colorectal cancer) with potential to translate to PDAC. Another upstream target is ERBB2/3, in which pan-ERBB inhibition with afatinib was preclinically shown to be synergistic with KRAS-G12D inhibition using MRTX-1133 in pancreas patient-derived organoids (92). CodeBreak101 (NCT04185883) is ongoing with a combination of sotorasib and afatinib for dual KRAS−ERBB inhibition. SOS1 is a RAS-guanine nucleotide exchange factor that turns “on” KRAS by facilitating GTP loading while SHP2 activates SOS1, making these attractive targets in the RAS pathway. Preclinical models have shown synergistic activity with dual inhibition of SHP2 and KRAS-G12C (93–95). There are ongoing trials (JAB3312 SHP2 inhibitor; NCT05288205) to examine the effect of targeting KRAS and SHP2 in patients with various malignancies that include PDAC. Targets downstream of KRAS being explored in other malignancies include MEK, RAF, and mTOR (84). In an ongoing trial in PDAC, avutometinib (RAF/MEK inhibitor) and defactinib (FAK inhibitor) are being used in combination with chemotherapy (NCT05669482). Preliminary results presented at the 2025 ASCO conference show tolerability with no dose-limiting toxicity and ORR ≥25% across five dose levels being evaluated (96). With the central role that KRAS plays in promoting an immunosuppressive tumor microenvironment in PDAC, inhibiting oncogenic KRAS mutants could increase the susceptibility of PDAC to immunotherapy as shown in preclinical studies (97). Animal studies have demonstrated that the basal state of PDAC cells, typically associated with chemoresistance, is particularly sensitive to KRAS inhibition (98, 99). This underlies the growing number of clinical trials combining KRAS inhibition with chemotherapy in PDAC (NCT06445062).

### KRAS-WT PDAC

In a minority of PDACs with no mutation in *KRAS* (5%–8%; of which about 40% have oncogenic alterations in the MAPK pathway), other actionable targets are more frequently present (100, 101). These include MMR deficiency; alterations in *BRAF*, *NF1*, and *ERBB2*; *FGFR* and *NTRK* fusions; and rearrangements in *NRG1*, *ALK*, *ROS1*, and *RET*. Several drugs targeting these oncogenic alterations have tumor-agnostic approvals, representing the predominant pathway for targeted therapies in this subset of PDAC tumors. The first positive randomized phase III trial (NOTABLE) in patients with *KRAS*-WT PDAC combined nimotuzumab (anti-EGFR) with gemcitabine versus gemcitabine alone in locally advanced or metastatic disease (91). The addition of nimotuzumab improved mOS to 10.9 months (95% CI, 5.6–16.3 months) in the gemcitabine/nimotuzumab arm versus 8.5 months (95% CI, 5.7–10 months) in the gemcitabine only arm; HR 0.66 (95% CI, 0.42–1.05). A trial to evaluate chemotherapy ± EGFR-targeted therapy (panitumumab) in RAS-WT PDAC has activated in the United States (NCT06998940).

A single-institution study found an alternative oncogenic driver in 60.3% (44/73 patients) of *KRAS*-WT PDAC (100). This underpins that importance of universal genomic testing of patients that present with PDAC, as it can identify potential targets. The proportion of patients with *KRAS*-WT PDAC is higher in patients younger than 55 years of age (100–102).

### HRD PDAC

The most frequently mutated core HR DNA damage repair genes in PDAC are *BRCA2*, *BRCA1*, and *PALB2*. HRD is defined by specific mutational patterns and genomic scars that arise from accumulation of defects in the genome due to a loss in the integrity of the homologous repair pathway. Examples are COSMIC 3, *BRCA* mutational, and unstable genome signatures (103, 104). PDAC with HRD display increased sensitivity to platinum and other DNA-damaging agents and are purportedly more immunogenic (105). The SHARON study (NCT04150042) is a phase I/II trial that takes advantage of the DNA damage repair deficiency in HRD tumors by treating with high-dose chemotherapy, after which patients are rescued with autologous stem cell transfer. Targeted agents that utilize the mechanism of synthetic lethality are also being evaluated in HRD tumors. Building on POLO, the APOLLO trial (NCT04858334) is a randomized phase II study investigating adjuvant olaparib in patients with resected pancreas malignancies (including acinar cell carcinoma) that have a pathogenic germline/somatic variant in *BRCA1/2* or *PALB2* and have completed standard perioperative therapy. Investigational strategies include combining synthetic lethal approaches with ICB in HRD tumors as in the POLAR pancreas cancer trial (NCT04666740) in which patients with platinum-sensitive disease with or without HRD mutations receive maintenance therapy consisting of pembrolizumab and olaparib as described previously (50). The SWOG S2001 is a randomized trial also investigating combination pembrolizumab and olaparib as a maintenance regimen in PDAC with germline *BRCA1/2* variants (NCT04548752; ref. 106). Mechanisms of resistance to PARPi involve restoring homologous recombination, loss of which is essential for synthetic lethality; for example, reversion mutations that restore the reading frame of *BRCA* mutations (107). Resistance also occurs via other mechanisms, including epigenetic modification, replication fork protection, and restoration of ADP-ribosylation. Interestingly, in pancreatic acinar cell carcinoma (PACC), HRD tumors occur with much higher frequency than seen in PDAC. Large genomic and clinical analyses found 23% to 48% of PACC had mutations in core HRD genes (108). With its rarity, PACC has typically been managed like PDAC; however, these studies emphasize the larger role of targeted therapy in PACC compared with PDAC.

## Immunotherapy and Therapies Targeting the Tumor Microenvironment

The tumor−immune microenvironment (TIME) of PDAC plays a critical role in its aggressive biology (109). The desmoplastic stroma forms a physically protective barrier. The TIME also contains cellular components that create an immunosuppressive environment, protecting it from immune surveillance and promoting EMT that enables dissemination of metastases (110, 111). Major components of the TIME include stellate cells, CAFs, myeloid-derived suppressor cells (MDSC), and tumor-associated macrophages (112). This role of the TIME in PDAC has led to significant interest in agents that target the TIME (113).

### Desmoplasia Targeting

Several components of the TIME desmoplastic stroma have been targeted, including matrix metalloproteinases (MMP), hyaluronidase, and collagenase. Collectively, these approaches have been unsuccessful (114–120). MMPs are important in the degradation of the extracellular matrix and expressed at a higher level in pancreas cancer compared with normal pancreas, making them an attractive therapeutic target (121). The MMP inhibitor tanamostat was found to be inferior to gemcitabine in metastatic PDAC (116); whereas marimastat did not improve survival when added to gemcitabine in patients with metastatic PDAC (117). Hyaluronan accumulates in the tumor microenvironment and impairs drug perfusion. Pegylated recombinant human hyaluronidase (PEGPH20) targets hyaluronan, but when combined with chemotherapy, led to poorer outcomes with FOLFIRINOX (114) and no increase in OS when added to gemcitabine/nab-paclitaxel (115). Ibrutinib modulates the TIME via antifibrotic and anti-inflammatory mechanisms but showed no improvement in OS or PFS when combined with gemcitabine/nab-paclitaxel (122). Interestingly, several of these studies noticed altered immune behavior within the tumor microenvironment in response to stromal reprograming by the targeted drugs. Newer therapeutic approaches target the TIME structure and immunomodulate concurrently (123, 124). Several such trials target the stromal-immune cross-talk pathway, especially CXCL12−CXCR4. A phase Ia study of LY2510924 (CXCR4 antagonist) in combination with anti–PD-L1 (durvalumab) in nine patients showed the combination was safe (125). The phase IIa COMBAT trial combined BL-8040 (a CXCR4 inhibitor) with checkpoint blockade (pembrolizumab) ± chemotherapy in patients with PDAC (126). Disease control rate of 34.5% was observed in the nonchemotherapy cohort and 32% in the cohort that received chemotherapy. Of note, BL-8040 increased CD8^+^ T-cell tumor infiltration while decreasing MDSCs and circulating regulatory T cells. Targeting focal adhesion kinase (FAK) has been shown to alter the TIME and inhibit pancreatic cancer cells (127). Defactinib, a FAK inhibitor, is being explored in combination with avutometinib and chemotherapy, as previously noted above (NCT05669482).

### Immunotherapy

ICB has revolutionized the treatment of several cancer types; however, PDAC is not among them and is considered the prototypic “cold” tumor. Anti–PD-1 (programmed cell death 1) and anti–CTLA-4 (cytotoxic T-lymphocyte–associated protein 4) drugs have been extensively studied in PDAC but found to provide no benefit as monotherapy, dual agents, or in combination with chemotherapy in unselected patients (128–131). The exception of response is the subset of PDACs that are dMMR/MSI-H (∼1%) or HRD (∼7%), mostly occurring in the setting of Lynch syndrome and rarely of somatic etiology (132, 133). Botensilimab, an Fc-enhanced anti–CTLA-4 antibody that optimizes T-cell priming and activation, extends the benefit of traditional anti–CTLA-4 to “cold” tumors (134). A phase I study combining botensilimab with the anti–PD-1 balstilimab in advanced microsatellite-stable tumors (NCT03860272) demonstrated clinical benefit, and the results in colorectal cancer showed promise (135). A follow-up randomized phase II trial to evaluate the efficacy of adding botensilimab to gemcitabine and nab-paclitaxel has completed recruitment in PDAC (NCT05630183). Acasunlimab (GEN1046) is a bispecific antibody designed to simultaneously stimulate T cells (agonistic 4-1BB) and inhibit checkpoint (anti–PD-L1). It showed promising antitumor preclinical results in pancreatic models (136), with a phase I/IIa clinical trial recently completed and the final results pending (NCT03917381).

Vaccines targeting RAS are under active development in PDAC. Ongoing studies are exploring peptide-based vaccines like the ELI-002 KRAS mutant–specific vaccine that uses an amphiphile modification to enhance lymph node delivery and enhance immune response. The phase I AMPLIFY-201 study of ELI-002 2P (targeting KRAS-G12D and G12R) significantly improved survival in the two-thirds of patients that were immune responders, defined as having at least a 9.17-fold increase in T-cell response (RFS HR, 0.12; 95% CI, 0.022–0.615; *P* = 0.0002; OS HR, 0.23; 95% CI, 0.063–0.854; *P* = 0.0099; ref. 137). This led to a randomized phase II trial with ELI-002 7P (targeting KRAS-G12D, G12R, 12V, 12A, 12C, 12S, and G13D) in patients with resected PDAC (NCT05726864), which has completed enrollment with final analysis pending. Another peptide KRAS vaccine (targeting KRAS-G12D, G12V, G12R, G12C, G12A, and G13D) with an immune adjuvant was combined with checkpoint blockade in a small study of patients with resected PDAC. This vaccine showed good immunogenicity with increased mKRAS-specific T-cell response (>5 fold increase in IFNγ-producing T cells) after vaccination (138), with a phase II trial underway (NCT05013216). These peptide vaccines are “off-the-shelf” vaccines, in addition to which there is ongoing evaluation of personalized vaccines in PDAC. The combination of high neoantigen quality and abundant CD8^+^ T-cell infiltrates was identified to correlate with longevity of patients with PDAC (139). In a phase I trial, individualized neoadjuvant vaccines (autogene cevumeran) synthesized from resected PDAC were administered in combination with atezolizumab (anti–PD-L1) and adjuvant FOLFIRINOX in 16 patients. Autogene cevumeran induced high neoantigen-specific T cells in 50% of the patients treated, known as responders, and they were also shown to have a longer median recurrence-free survival compared with nonresponders (NR vs. 13.4 months; *P* = 0.007; HR, 0.14; 95% CI, 0.03–0.59; ref. 140). A randomized phase II trial in patients with resected PDAC treated with FOLFIRINOX ± the individualized vaccine autogene cevumeran is ongoing (NCT05968326). Additional immunomodulatory therapeutic targets include immune stimulators/agonists (TLR4, interferon), T-cell receptor therapies, ADCs, and novel immune checkpoint inhibitors (LAG3, TIGIT, and newer-generation CTLA-4 inhibitors).

### Novel Immune-Mediated Combinations

The TIME allows PDAC cells to combine metabolic, physical barrier, and immunosuppression pathways to create a tumor-promoting ecosystem. Therapies that have focused on each of these individual components have been largely unsuccessful. In addition to chemoimmunotherapy regimens (141), novel combinations that simultaneously target multiple pathways seem promising. Immunotherapy regimens that combine immune activation (CD-40 agonist) with inhibition of immune suppression (ICB) are being explored (NCT03329950). Within the growing role of bispecific agents in cancer therapy, an agent that is showing promise is AFM24. This is a T cell–independent bispecific innate cell engager that binds CD16A on NK cells/macrophages and EGFR on tumor cells (142). A phase II study combining AFM24 with atezolizumab in several tumor types, including pancreatic cancer, is underway (NCT05109442). RO7122290 is a bispecific fusion protein with 4-1BB (CD173 ligand) that costimulates T cells and an FAP-binding site to target the drug to FAP, which is enriched in the PDAC microenvironment. A first-in-human phase I study demonstrated safety, immune activation, and tumor response as monotherapy or in combination with atezolizumab (143). Building on the success of bispecific antibody agents are trispecific drugs that have multiple concurrent targets. HPN536 is a trispecific T-cell engager that targets mesothelin, enriched in the PDAC tumor microenvironment and associated with poorer prognosis. It binds to MSLN on tumor cells, CD3 on T cells, and serum albumin to extend plasma half-life (144); a phase I/IIa study was recently completed, with the results pending (NCT03872206).

## Selected Populations for Focus in PDAC

Significant disparities in incidence and outcomes of PDAC exist among different populations by age, sex, geography, culture, socioeconomic status (SES), and more. There is a rising incidence of PDAC in younger individuals that was localized particularly to birth cohorts in the 1980s and 1990s; exemplified by a 2.6-fold higher PDAC incidence in the 1990 cohort compared with the 1955 cohort (145). In those younger than 55 years old, women were found to be disproportionately affected compared with men (HR, 1.93; 95% CI, 1.57–2.28; *P* < 0.001; ref. 146). It remains unclear why this has occurred, with multiple hypotheses being explored. In the United States, Black/African Americans (AA) have a 40% to 90% higher incidence and 20% worse survival than other races (147–150). Several factors have significant overlap—such as SES, geography, and race—that pose a challenge in deciphering the contribution of biology versus environment in these disparities. Social determinants of health that affect one’s environment contribute significantly to these disparities, as noted by a lower rate of surgical resection and reduced access to high volume centers in Hispanic and AA patients compared with non-Hispanic White counterparts (151). Genetic and epigenetic differences among different groups may also contribute to the observed disparities, particularly as these differences are understudied (152). Disease biology may contribute to differences observed between patient groups. Enrichment of certain biologic characteristics within a group provides an opportunity to better understand PDAC. The enrichment of *BRCA*-mutated PDAC in Ashkenazi Jewish populations is an example of how biomarker enrichment can lead to therapeutic targeting. These differences among various groups highlight the need for more studies in these populations. Studying disparities seen in underrepresented patient population groups can be challenging due to smaller numbers enrolled in studies. Enhancing diversity in clinical trial participation by reducing restrictions in eligibility criteria and prioritizing community engagement represents an opportunity to improve access to care.

## Conclusion

Chemotherapy is established as a cornerstone therapeutic paradigm, with multiple randomized trials endorsing OS improvements in all stages of PDAC. Nonetheless, progress to date has been modest and incremental with advancement in outcome urgently needed. The future of systemic therapy for PDAC focuses on the integrated use of targeted and immunomodulatory agents. Drugs that target mutant (K)RAS are expected to have significant impact in PDAC and are predicted to change the standard of care in the proximate future. Promising therapeutics targeting KRAS include direct inhibitors, degraders, and immunomodulatory approaches. ICB is largely ineffective in PDAC due to immunosuppression and other complexities. The development of drugs that utilize novel immune mechanisms and the use of regimens which target multiple steps in the immune and stromal environment provide an opportunity for surmounting intrinsic immune resistance. The authors are optimistic that we are on the cusp of witnessing substantive improvements in outcome in PDAC as we integrate the learnings from the pathobiology of this disease into innovative clinical trials and new therapeutic approaches.
